# Fluorescent nuclear track detectors for alpha radiation microdosimetry

**DOI:** 10.1186/s13014-018-1034-x

**Published:** 2018-06-07

**Authors:** J. J. M. Kouwenberg, H. T. Wolterbeek, A. G. Denkova, A. J. J. Bos

**Affiliations:** Radiation, Science & Technology, Delft University of Technology, Mekelweg 15, Delft, The Netherlands

**Keywords:** Fluorescent nuclear track detector, FNTD, Alpha radiation, Microdosimetry, Spheroid, Alpha radionuclide therapy

## Abstract

**Background:**

While alpha microdosimetry dates back a couple of decades, the effects of localized energy deposition of alpha particles are often still unclear since few comparative studies have been performed. Most modern alpha microdosimetry studies rely for large parts on simulations, which negatively impacts both the simplicity of the calculations and the reliability of the results. A novel microdosimetry method based on the Fluorescent Nuclear Track Detector, a versatile tool that can measure individual alpha particles at sub-micron resolution, yielding accurate energy, fluence and dose rate measurements, was introduced to address these issues.

**Methods:**

Both the detectors and U87 glioblastoma cell cultures were irradiated using an external Am241 alpha source. The alpha particle tracks measured with a Fluorescent Nuclear Track Detector were used together with high resolution 3D cell geometries images to calculate the nucleus dose distribution in the U87 glioblastoma cells. The experimentally obtained microdosimetry parameters were thereafter applied to simulations of 3D U87 cells cultures (spheroids) with various spatial distributions of isotopes to evaluate the effect of the nucleus dose distribution on the expected cell survival.

**Results:**

The new experimental method showed good agreement with the analytically derived nucleus dose distributions. Small differences (< 5%) in the relative effectiveness were found for isotopes in the cytoplasm and on the cell membrane versus external irradiation, while isotopes located in the nucleus or on the nuclear membrane showed a substantial increase in relative effectiveness (33 – 51%).

**Conclusions:**

The ease-of-use, good accuracy and use of experimentally derived characteristics of the radiation field make this method superior to conventional simulation-based microdosimetry studies. Considering the uncertainties found in alpha radionuclide carriers in-vivo and in-vitro, together with the large contributions from the relative biological effectiveness and the oxygen enhancement ratio, it is expected that only carriers penetrating or surrounding the cell nucleus will substantially benefit from microdosimetry.

## Background

The interest in alpha radionuclide therapy remains on the rise [1–5], partly for its ability to deal with (micro-) tumours resistant to gamma and beta radiation attributed to the high LET of alpha particles [6, 7]. Microdosimetry for alpha particles deals with the determination of the absorbed dose distribution in cell nuclei, the so called nucleus dose distribution (NDD) [8]. The high LET of alpha particles, and therefore the relative low fluence per absorbed dose, in combination with the stochastic nature of radiation, leads to large variances in the NDD. This introduces anomalies when comparing the survival of cells in various alpha radiation fields [9]. Microdosimetry methods already exist for alpha radiation [8, 10–12]. However, most of these methods require far-reaching assumptions regarding the radiation field and cell geometries, which have a negative impact of the accuracy and simplicity of these methods [9, 13, 14]. This paper utilizes a novel type of detector called the Fluorescent Nuclear Track Detectors (FNTD) [15, 16], which allows for robust measurement of individual alpha particles with great accuracy [17, 18]. Using these detectors, the point of entrance, direction and energy of alpha particles tracks can be measured at sub-micrometer resolution [19, 20]. This detailed knowledge of the radiation field can be used to calculate the energy deposited in cell nuclei with relative ease and without the need for assumptions regarding the radiation field characteristics or geometry. The resulting microdosimetric spectra can be of great value for both cell survival studies as well as DNA damage and repair studies.

Alpha microdosimetry is also an area for which the criteria for application are not always clear, since very few studies exist that compare the effects of localized energy deposition of alpha particles for various scenarios [9, 10, 21]. Since microdosimetry is often cumbersome, it is important to know under what conditions to apply and when to limit the time spend on microdosimetry during the design and evaluation of alpha radionuclide carriers (ARC). This study therefore extends the knowledge obtained using the FNTD experiments to simulation of virtual spheroids. Spheroids, a type of 3D cell culture that mimics the behavior of (micro-) tumors, are a better model to verify the effectiveness of ARCs than monolayers for alpha radiation therapy, both from a dosimetry and a radiobiology point of view [22]. Simulations for various types of sub-cellular alpha radionuclide distributions and alpha particle energies were performed to clarify the effect of NDD-based microdosimetry on the expected survival.

## Methods

### Irradiation setup

An irradiation setup described in a previous publication [23] was built specifically for FNTD alpha (micro-) dosimetry. A 1.1 cm diameter 394 MBq Am241 source supplied by Czech Metrological Institute was placed inside a 3D printed honeycomb collimator and mounted on linear x,y-stages. The 1800 μm thick honeycomb collimator restricted the vertical angle of alpha particles to 45 degrees to yield sufficient penetration in the FNTD for proper track measurement. The source was moved in the x,y-plane to uniformly irradiate areas up to 9 times the area of the source. Both cells and FNTDs were irradiated 5.0 ± 0.1 mm above the source, yielding a dose rate of 4.15 mGy/s ± 5.3% in the cell layer. This dose rate was checked with an independent measurement with an interpolation chamber.

### Cell survival

U87 Glioblastoma cells were cultured in Dulbecco’s Modified Eagle Medium supplemented with 10% Fetal Bovine Serum and 1% Penicillin-Streptomycin. 35 mm lumox® dishes (Sarstedt AG & Co., Nümbrecht, Germany) were modified to hold a 1.4 μm mylar film bottom, required for sufficient penetration of the alpha particles into the cell layer. The modified dishes were irradiated to 10 kGy from a Co-60 source for sterilization. U87 cells in culture flasks were resuspended using trypsin, pipetted repeatedly to avoid cell clumps and counted using a digital cell counter, after which 60.000 cells were transferred to mylar dishes. The cells were allowed to settle overnight and irradiated to 0, 125, 249, 498, 747 and 996 mGy (± 5.3%) [23]. To minimize stress induced pH changes following atmospheric exposure, cells were irradiated in medium with 20 mM HEPES and each mylar dish was exposed to ambient air for an equal amount of time. After irradiation, 1900 – 5000 cells per well, depending on the expected survival, were transferred from the mylar dish onto 3 wells in a 24-well plate. The 24-well plates were incubated for 7 days after which the number of surviving cells were counted using the SRB assay [24]. The procedure was performed twice to improve reliability, which was deemed sufficient for the proof-of-principle of this work.

### Cell geometry measurement and segmentation

U87 cells were transferred onto glass cover slips and allowed to settle overnight, after which they were fixated for 15 min at room temperature using 3.7% formaldehyde. The nucleus and cytoplasm were stained using respectively DAPI and Alexa Fluor 488 Phalloidin. A total of 15 cells were imaged using a Leica SP5 with a 63 × 1.40 NA oil objective and a 96 × 96 × 430 nm^3^ (x,y,z) voxel size (after refractive index mismatch correction [25]). The measured slice thicknesses were corrected for the difference in reflective index [25]. The nuclei and cytoplasm in the images were segmented via thresholding using Fiji [26] yielding an image stack with 8-bit pixel values of 0, 182 and 201 for respectively the background, cytoplasm and nucleus.

### Analytical estimation of survival based on the nucleus dose distribution

The number of particles hitting a nucleus is discreet. The probability distribution of a cell receiving a dose *z* can therefore be calculated from the Poisson distribution of the number of particles hitting the cell nucleus and a function describing the energy deposition by a single track, the so called single hit nucleus dose distribution (SHNDD). Note that the NDD and SHNDD are both sometimes referred to as the specific energy. In this work, NDD and SHNDD will however be used for clarity. Let *q* be the mean dose of the SHNDD and therefore represent the average dose induced by a single particle in the nucleus, so that the number of tracks per nucleus, given an average dose $\overline{z}$, follows from the Poisson distribution via [27]:

1 $$ p\left(i,\lambda =\overline{z}/q\right)=\raisebox{1ex}{${\lambda}^i{e}^{-\lambda }$}\!\left/ \!\raisebox{-1ex}{$i!$}\right. $$

Where *p* is the Poisson distribution, giving the probability of *i* particles passing through a cell nucleus given an average number of particles *λ* hitting the cell nucleus at an average dose $\overline{z}$. The nucleus dose probability distribution for *i* particles hitting the cell nucleus can then be constructed via *i* convolutions of the SHNDD *f*^*i* = 1^(*z*) [10], which is described in Laplace space by:

2 $$ {f}^i(z)={\mathcal{L}}^{-1}\left\{\mathcal{L}{\left\{{f}^{i=1}(z)\right\}}^i\right\} $$

The SHNDD *f*^*i* = 1^(*z*) is in this case obtained using either experimental methods or simulation. Let $f\left(z,\overline{z}\right)$ be the NDD, representing the probability of a cell nucleus receiving a dose z given the average dose $\overline{z}$. One can find this probability by summing the contributions of the nucleus dose probability functions for *i* particles hitting the cell nucleus, *f*^*i*^(*z*), where *i* ranges from 0 to *N* particles. *N* is chosen as a very large number so that the likelihood of a cell nucleus being hit by *N* particles is very small. The NDD distribution then follows from [27]:

3 $$ f\left(z,\overline{z}\right)=p\left(i,\lambda =\overline{z}/q\right)\delta (z)+\sum \limits_{i=1}^Np\left(i,\lambda =\overline{z}/q\right)\ {f}^i(z) $$

*δ*(*z*) is here the Dirac delta function, taking a value of 1 when the dose *z* is 0, and taking a value of 0 everywhere else. Now let the survival of a cell colony at absorbed dose *x* be given by *S*(*x*) = *e*^−*αx*^. Note that the term *α* is simply the first constant in the linear-quadratic equation for cell survival, i.e. we neglect the quadratic term. The cell survival based on the nucleus energy deposition distribution is then obtained by integrating over the NDD [10]:

4 $$ {S}_z\left(\overline{z}\right)={\int}_0^{\infty }f\left(z,\overline{z}\right)\bullet {e}^{-{\alpha}_z\ z} dz $$

Where *α*_*z*_ is the microdosimetric survival slope. The survival ${S}_z^C\left(\overline{z}\right)$ for a collection of cells then follows from the summation of eq. (4) for the collection *C*:

5 $$ {S}_z^C\left(\overline{z}\right)=\frac{1}{M}\sum \limits_{l=1}^M{S}_{z,l}\left(\overline{z}\right) $$

where *l* = 1. . *M* represent the *M* cells in collection *C* with corresponding average absorbed dose induced by a single particle *q*_*l*_. Using the previously mentioned survival equation, the expected survival from the classical approach can be calculated using the absorbed dose:

6 $$ {S}_D\left(D={\overline{z}}_n\right)={e}^{-{\alpha}_DD} $$

Where the survival slope *α*_*D*_ is the experimentally determined survival slope, which is only valid for the radiation field in which it was measured. Since the survival predicted via microdosimetry and the absorbed dose must be equal for the same radiation field, the microdosimetric survival slope *α*_*z*_ can be obtained by equating Eq. (5) to eq. (6). This relation can be rewritten to:

7 $$ {\alpha}_D\bullet D+\mathit{\ln}\left[{S}_z^C\left(D={\overline{z}}_n\right)\right]=0 $$

Since this equation cannot be solved analytically, *α*_*z*_ must be acquired numerically, in this case using the non-linear least squares algorithm as found in the software package R version 3.2.2 [28]. This optimization will attempt to solve the following equation:

8 $$ \underset{\alpha_z\in \mathbb{R}}{\min}\sum \limits_j{\alpha}_D\bullet {D}_j+\mathit{\ln}\left[{S}_z^C\left({D}_j={\overline{z}}_{n,j}\right)\right] $$

where *D*_*j*_ are the absorbed doses used during the biological survival experiments. For these experiments, the uncertainty in *α*_*z*_ is assumed to follow from the derivative of *α*_*z*_ to *α*_*D*_ and the experimental uncertainty of *α*_*D*_, i.e. the standard error propagation.

### FNTD irradiation, read-out and analysis

**Fig. 1:**
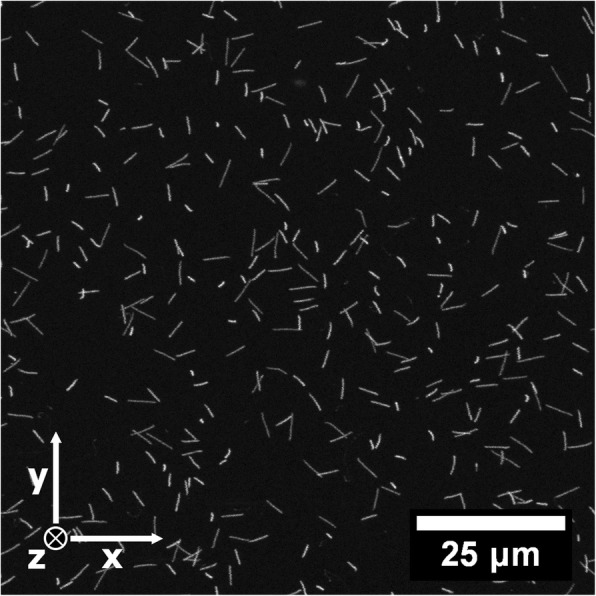
A 2D maximum intensity projection of the fluorescence measured in a 100 × 100 × 12 μm^3^ (x,y,z) volume with a confocal laser scanning microscope in a FNTD after irradiation with alpha particles. The apparent length of the tracks is related to the angle of incidence of the alpha particles. Individual tracks were analyzed using in-house build software, yielding the start- and endpoint, direction, relative scattering, fluorescence intensity and energy for each track [20]

Eight FNTDs were irradiated at specific locations in the irradiation field to yield the best representation of irradiated area [23]. The 100 × 100 × 15 um^3^ (x,y,z) center region of each FNTD was read-out using a Leica SP5 with Avalanche Photo Diode (APD), a 63 × 1.4 NA oil objective and a 96 × 96 × 666 nm^3^ (x,y,z) voxel size (after refractive index mismatch correction [25]). FNTDs allow for the measurement of individual alpha particle tracks at a sub-micron resolution. An example of alpha tracks measured in a FNTD is given in Fig. 1. For each track, the point of entrance, direction and energy upon entering the FNTD was determined. The energy resolution was determined in a previous publication at approximately 100 – 200 keV [20]. Tracks ending in the outer 1 μm rim of the image stack were removed since these tracks were likely to come to a halt outside the field of view, leading to incorrect track reconstruction.

### FNTD specific energy calculation

**Fig. 2:**
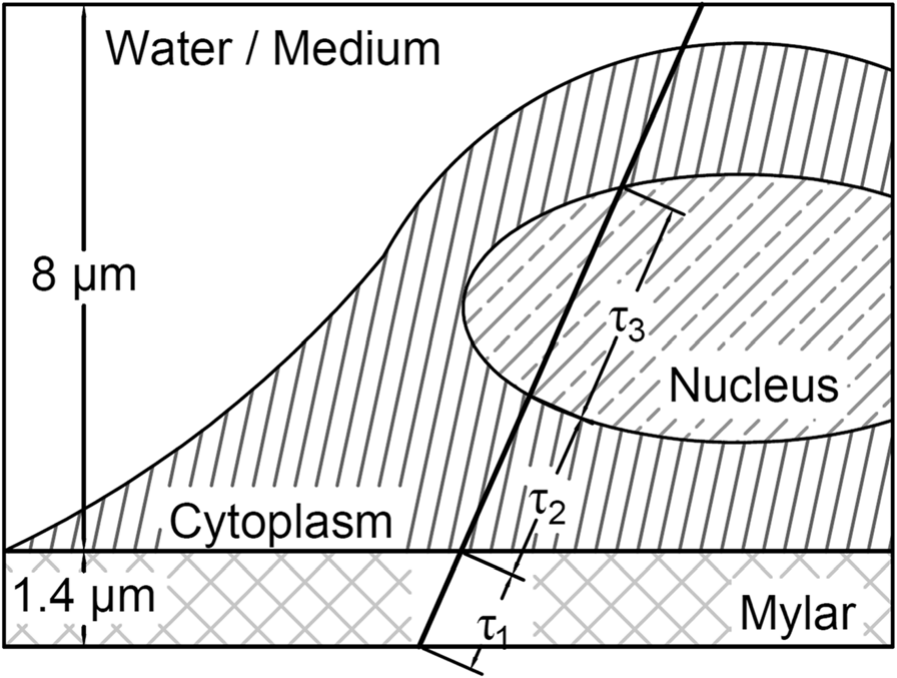
Illustration of an alpha track passing through a cell. Path lengths through the various media are indicated by τ_1_ .. τ_3_

In order to calculate the NDD of a cell, the segmented image stack was placed virtually in the radiation field, measured using the FNTDs. Given the initial direction and energy of a track, the dose deposited in a cell nucleus (Fig. 2) could be calculated by combining the path length through the cell nucleus (τ_3_) and the particle’s LET in water [29], corrected for the energy loss during traversal of the mylar foil (τ_1_) and the cytoplasm (τ_2_). The total dose deposited *D*_*total*_ by the collection of tracks (*N*_*total*_=3348 tracks) in a 8 μm water layer (the average height of the measured cells) above the 1.4 μm mylar foil was calculated similar to an earlier publication [23]. The dose deposited in the cell nucleus, given an absorbed dose $\overline{z}$, was calculated by randomly selection of *N* tracks (eq. (9) and calculating the dose deposited in the cell nucleus by tracks hitting the nucleus.

9 $$ N=\frac{\overline{z}}{D_{total}}\ {N}_{total} $$

By repeating this process many times, each time with a different random selection of tracks, the NDD $f\left(z,\overline{z}\right)$ as function of the absorbed dose $\overline{z}$ could be obtained. Both the cell nucleus and cytoplasm were assumed to have the stopping power and density of water for the dose calculations.

### Spheroid microdosimetry simulation

**Fig. 3:**
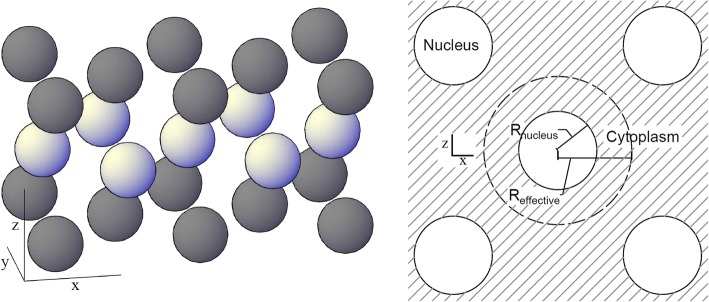
(left) 3D view of the virtual U87 nuclei in a HCP geometry. The dark and light grey spheres represent nuclei in respectively the even and uneven layers of the geometry. (right) 2D view of the hexagonal close packing geometry. The effective radius is indicated by R_effective_. Note that the striped border is the not the cell membrane and that the cytoplasm (lines) fills all the space between the nuclei (solid circles) in these simulations. The cell membranes have not been drawn in this illustration

Basic simulations of alpha emitting isotopes in U87 glioblastoma spheroids were performed to evaluate the effect of NDD-based microdosimetry on the expected survival. Virtual spherical U87 cells, with cytoplasm and nucleus volumes obtained from the cell images, were stacked using the Hexagonal Close Packing (HCP) geometry (Fig. 3, left). For these simulation, it was assumed that there would be no interstitial fluid in the spheroid. Since the HCP geometry has a packing efficiency of *η* ≈ 0.740, free space would remain between the cells when the geometry would be built using the radius of the cell as border between the spheres. By choosing a smaller radius of ${R}_{effective}\approx \sqrt[3]{0.740}\ {R}_{cell}$ as ‘border’ of the spheres in the geometry, the cytoplasm fills up all the volume between the nuclei, leaving zero interstitial fluid between the cells (Fig. 3, right).

Four different spatial distributions of isotopes were chosen to represent various ARCs: ARCs on cell membrane [30], ARCs evenly distributed in the cytoplasm [31, 32], ARCs on the nuclear membrane and ARCs evenly distributed in the nucleus [33]. The ARC distributions are here forth referred to as scenarios. During spheroid simulation, the origin (based on the given scenario) and direction of alpha particles originating from isotope decay events were randomly chosen. When the simulated particle hits the nucleus located at the center of the virtual spheroid, the path lengths through the spheroid and nucleus located in the center were calculated together with dose deposited in the nucleus and cytoplasm [29]. Simulations were performed for alpha particle energies between 4000 and 20.000 keV, and the HCP geometry was expanded to accommodate for the maximum range of the respective alpha particle energy. Each simulation was repeated till a total of 20.000 particles hitting the center nucleus was found. The statistical uncertainties of the mean absorbed doses in cell nuclei in these simulations were below 1.5%. The expected survival in the spheroid was calculated based on the SHNDD as described in an earlier section, together with the absorbed dose in the spheroid. Since this article only focusses on the significance of NDD-based microdosimetry, the dependency of the RBE on the LET was ignored in the survival calculations but is stated in the discussion of the results afterwards.

**Fig. 4:**
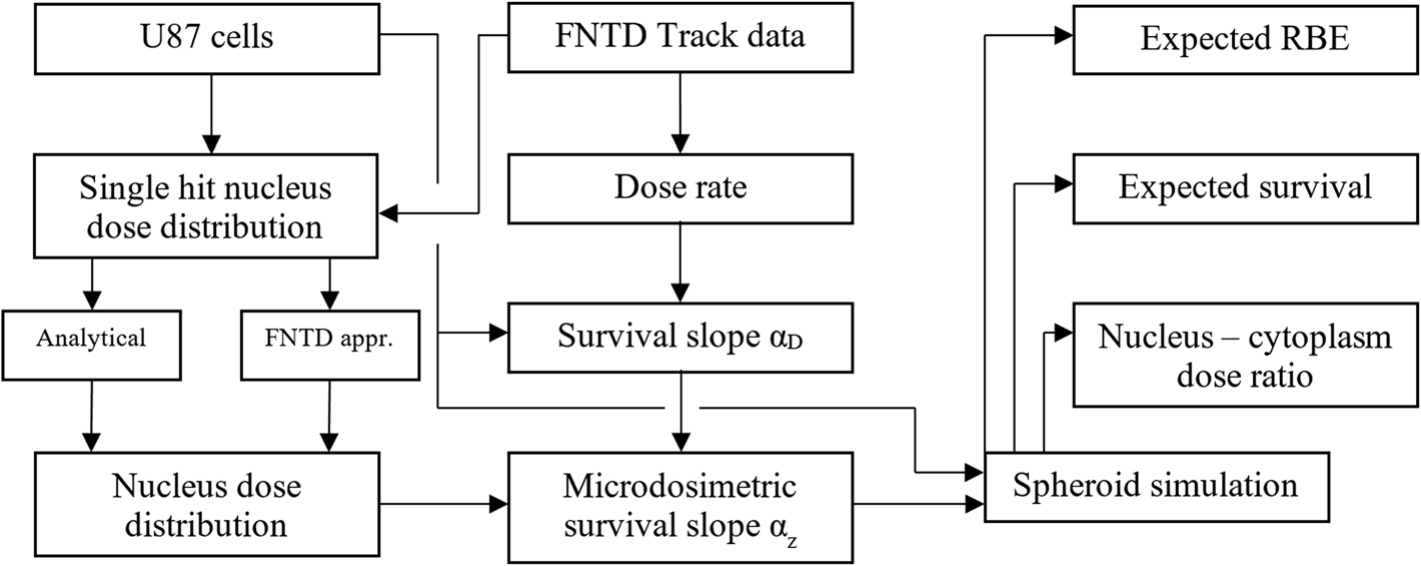
A diagram showing the work flow of this method, including the spheroid simulations that were performed using the parameters obtained using the FNTDs and U87 glioblastoma cells

An overview of the complete workflow for this method, from the U87 glioblastoma cells and measured tracks in the FNTD to the values obtained with the spheroid simulations, is shown in Fig. 4.

## Results and discussion

### Cell survival and geometry

**Fig. 5:**
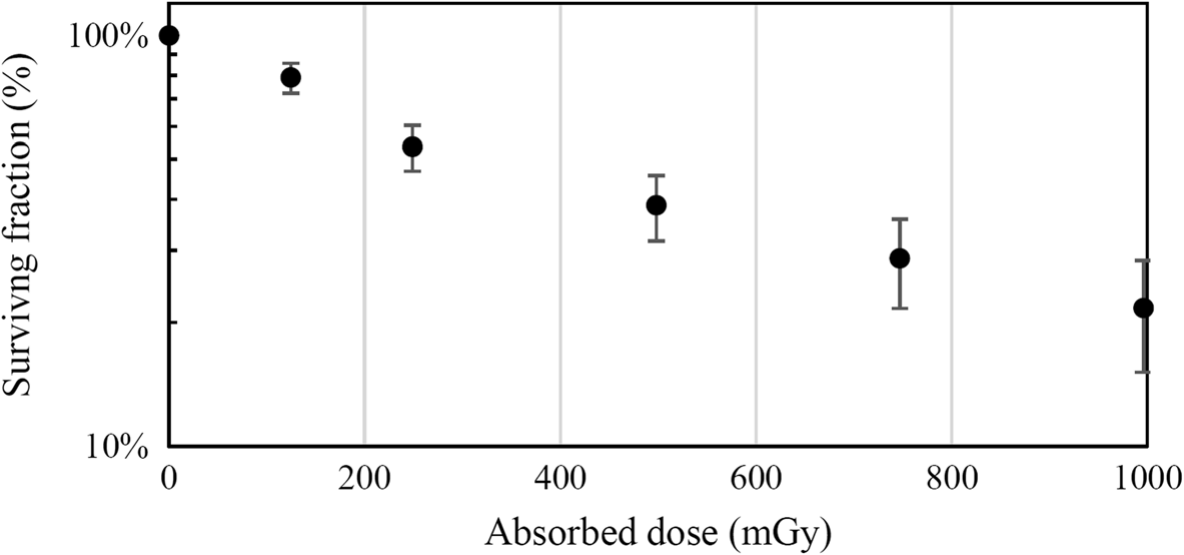
Measured surviving fraction as function of dose for U87 cells irradiated with an external Am241 source

The measured surviving fraction during the external irradiation, compared to the non-irradiated control, is given in Fig. 5. Fitting eq. (6) for *α*_*D*_ on the data yields *α*_*D*_ = 1.66 ± 0.13 Gy^− 1^. The relatively high uncertainty was partly caused by the many steps involved in transferring cells in and from the fragile irradiation dishes and the uncertainty in absorbed dose.

**Fig. 6:**
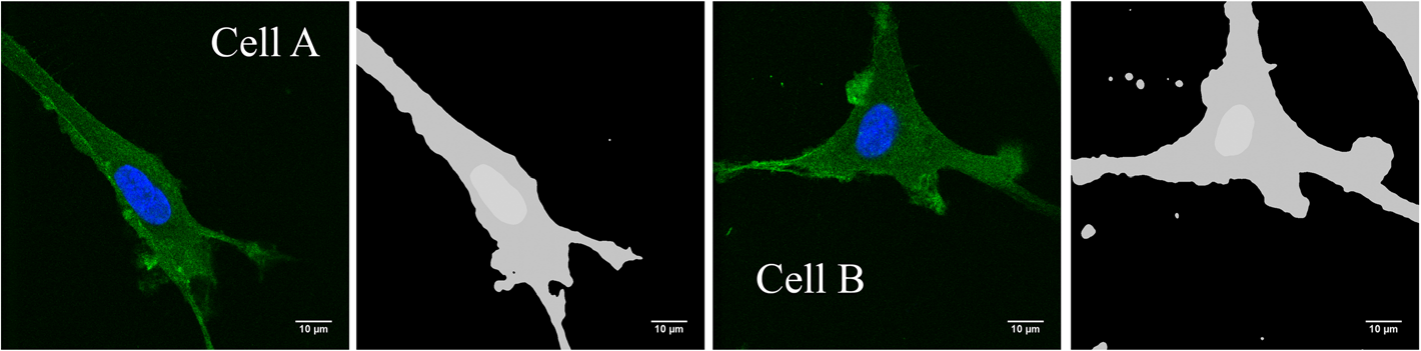
Examples of two U87 cells (cell A, left, and cell B, right) selected from the collection of imaged cells, stained for the cytoplasm (green/dark grey) and nucleus (blue, light grey). Image scans are given on the left with the corresponding segmentation on the right

**Fig. 7:**
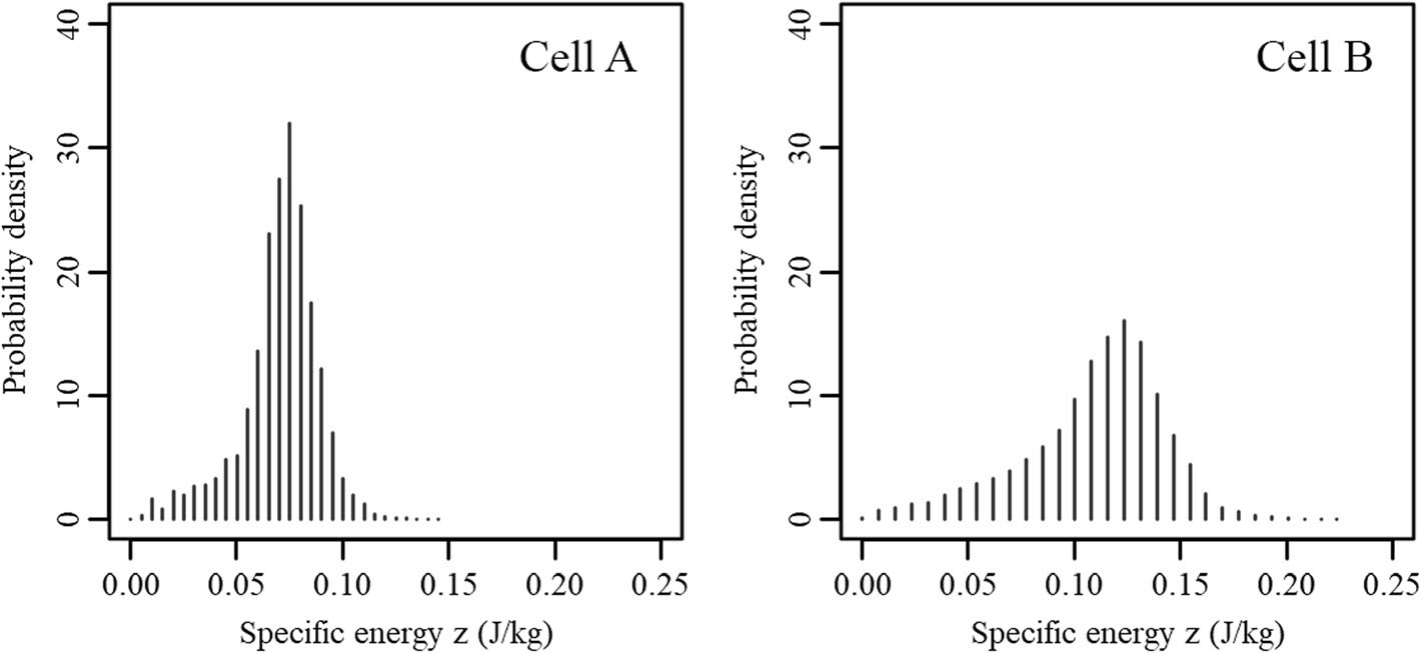
Nucleus dose distribution of nuclei hit by a single alpha particle while irradiated using an external Am241 source for cell A and B, respectively given in the left and right figure

**Fig. 8:**
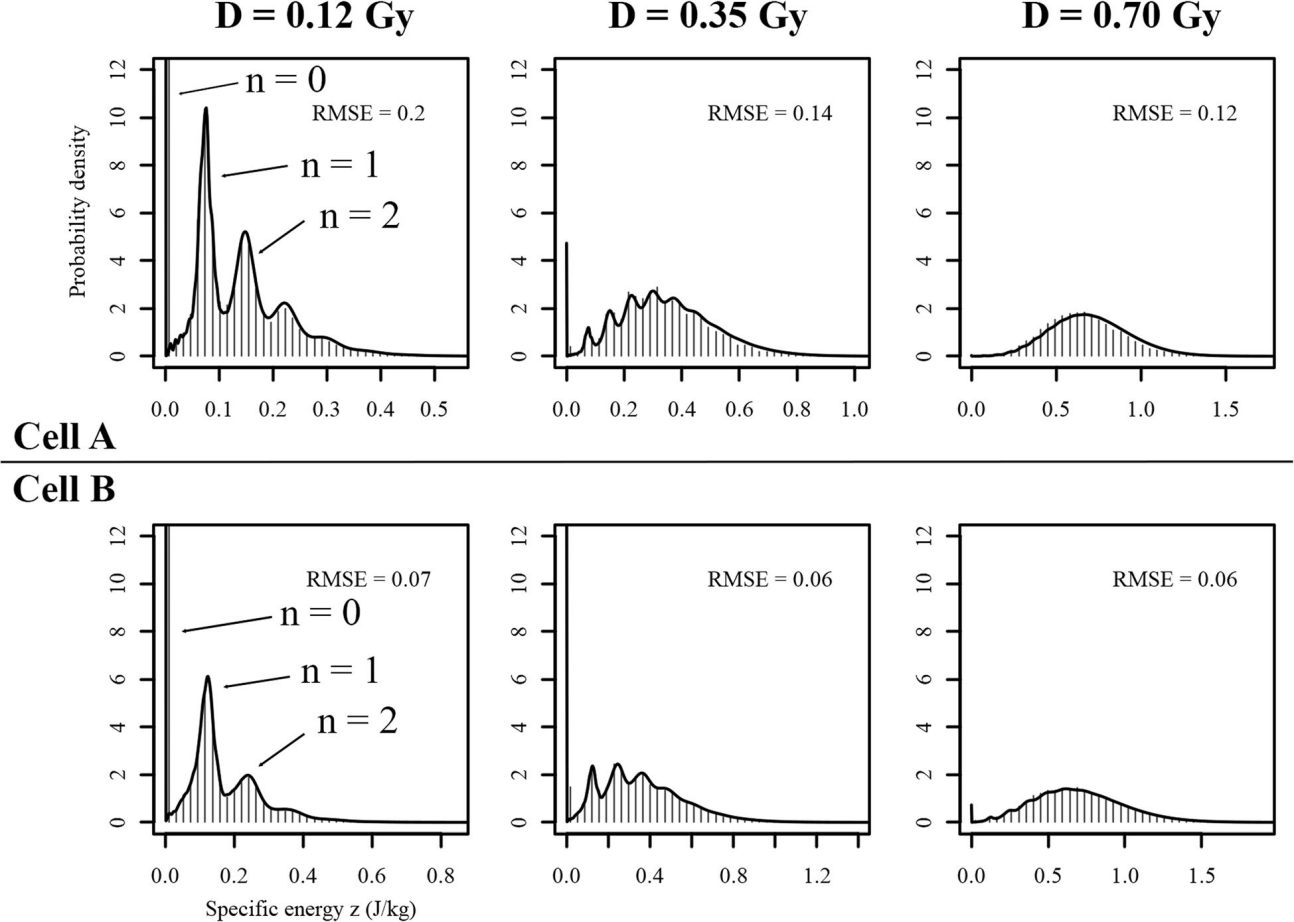
Nucleus dose distribution of cell nuclei at absorbed doses between 0.12 and 0.70 Gy. The histogram represents the distributions based on the FNTD approach, while the black line is given by the analytical method based on the single hit distributions given in Fig. 7. Some of the peaks are indicated by the number of particles (n) hitting the nucleus that led to the formation of the respective peak. The vertical line at z = 0 represents the probability that a nucleus is not hit at all (*n* = 0). The RMSEs between the FNTD and analytical approach are given in the top right of the graphs

The middle slices of the 3D images of 2 out of the 15 imaged cells (designated as cell A and cell B), with corresponding cytoplasm/nucleus segmentation, are shown in Fig. 6. The average nucleus volume of the 15 cells was 740 ± 150 μm^3^. While the displayed cells serve as examples for the NDD in Fig. 7 and Fig. 8, the microdosimetry-based survival calculations involved all 15 cells.

### Cell volume uncertainty

The imaging resolution was chosen so that the voxel size approximated half of the full width half maximum (FWHM) of the excitation spot, meaning that excitation spot was not solely confined to the imaged voxel and fluorescent dye in neighboring voxels would yield a (weakened) fluorescence response in the imaged voxel. The edge of the nucleus-cytoplasm border was visible as a smooth fluorescence intensity transition stretching approximately 2-3 voxels. The edge of the nucleus was chosen as the 50% fluorescence intensity decrease line (compared to the center of the nucleus). Since it was not possible to verify this assumption, a 1 voxel systematic uncertainty can be assumed. Given a voxel volume of 3.96E6 nm^3^ and an average nucleus volume of 740 μm^3^, the average nuclei consisted of 1.87E5 voxels, which translates to an average radius of 39 voxels (assuming a spherical nucleus volume). A systematic over- or underestimation of the nucleus edge by 1 voxel would therefore result in a relative error in nucleus volume of 5.1%. At the time of writing, it not possible to verify this number. It was therefore not included in the uncertainty calculations in this work. The reader is however advised to keep this possible source of uncertainty in mind when performing microdosimetry calculations and experiments.

### Nucleus dose distribution

The SHNDD, given as probability densities, calculated using the FNTDs for the cells displayed in Fig. 6 are given in Fig. 7. The means of the SHNDD were respectively 0.070 and 0.109 J/kg per particle. The differences in spread and mean are the result of differences nucleus size and shape and the thickness of the cytoplasm layer between the mylar sheet and the cell nucleus. The average energy deposited by an alpha particle passing through the collection of 15 cell nuclei was determined at 115 ± 10 keV.

The NDD for absorbed doses between 0.12 and 0.70 Gy for the two cells are given Fig. 8. The histograms represent the FNTD approach for NDD calculation for the indicated mean absorbed dose *D* in the cell culture, while the black lines indicate the NDD based on the analytical approach (eq. (3) and the single hit distributions given in Fig. 7. The distinct peaks in the distributions are due to the discreet nature of the number of particles hitting the nucleus and are clearly visible up to 0.35 Gy. When the absorbed dose, and therefore the average number of particles hitting the cell nucleus, increases, the distribution becomes more and more Gaussian like, which is conform the Poisson distribution at large means. Both the calculated histograms and analytically derived NDD were in close agreement, as indicated by the root mean squared errors (RMSE) between the two approaches.

### Monolayer expected survival

**Fig. 9:**
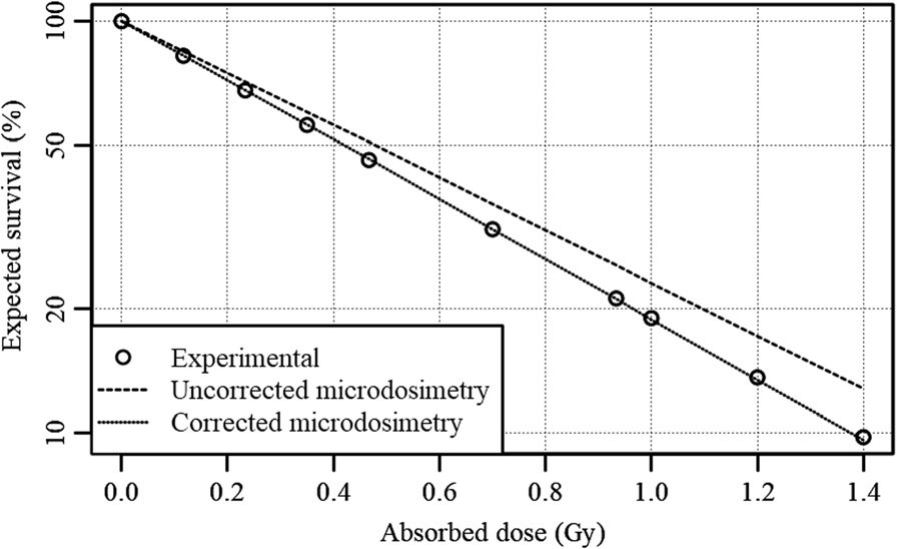
Expected survival based on the microdosimetry, both with and without survival slope correction, of externally irradiated U87 cells compared to experimental data

Using the NDD given in the previous section, the microdosimetry-based survival could be estimated with and without correction for the survival slope *α*_*z*_. The respective estimated survival curves are given in Fig. 9. Fitting of *α*_*z*_ using *α*_*D*_ = 1.66 ± 0.13 Gy^− 1^ and eq. (7) led to *α*_*z*_ = 1.92 ± 0.15 Gy^− 1^. Note that the possible systematic error in the nucleus volume estimation (5.1%) is not included in the uncertainty of *α*_*z*_. The microdosimetric approach without survival slope correction yielded a 13% underestimation in radiosensitivity, which signifies the importance of correct usage of the primary survival slope. The obtained survival slope *α*_*z*_ was used for further calculations in spheroids. The exact uncertainty of *α*_*z*_ beyond the uncertainty inherited from *α*_*D*_ is not entirely known. Contributions from cell geometry alteration due to fixation, errors in cell segmentation and others can each have a contribution to the uncertainty and require further investigation to better quantity the uncertainty of *α*_*z*_. However, it can be reasonably assumed that these uncertainties found in the FNTD will be smaller than uncertainties originating from the far-reaching assumptions made in modern microdosimetry simulation studies regarding the radiation field and the cell geometries. Note that while the used sample size of 15 cells was deemed sufficient for this proof of principle and the spheroid simulations, future studies looking to correlate microdosimetric parameters with biological indicators should increase the sample size to better reflect the whole culture population.

### Spheroid simulation

**Fig. 10:**
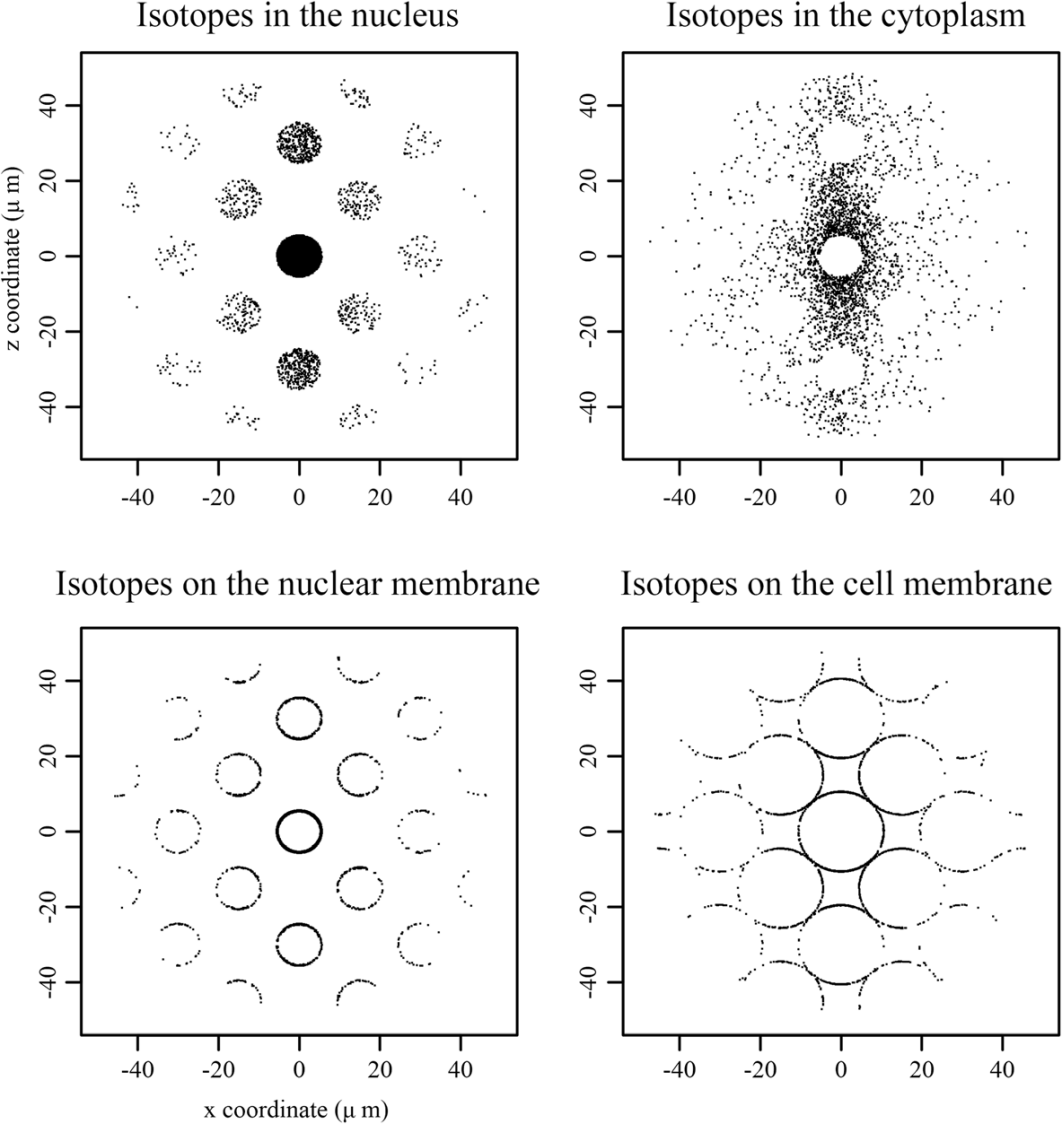
Spatial distributions of Am241 decay events which created alpha particles that deposited energy in the center nucleus for various spatial distributions of isotopes visualized in 2D. Events within Y = ± 2 μm were selected to create a 2D representation of the 3D distribution, which means that spheres in 3D are visible as circles in the 2D figure

**Fig. 11:**
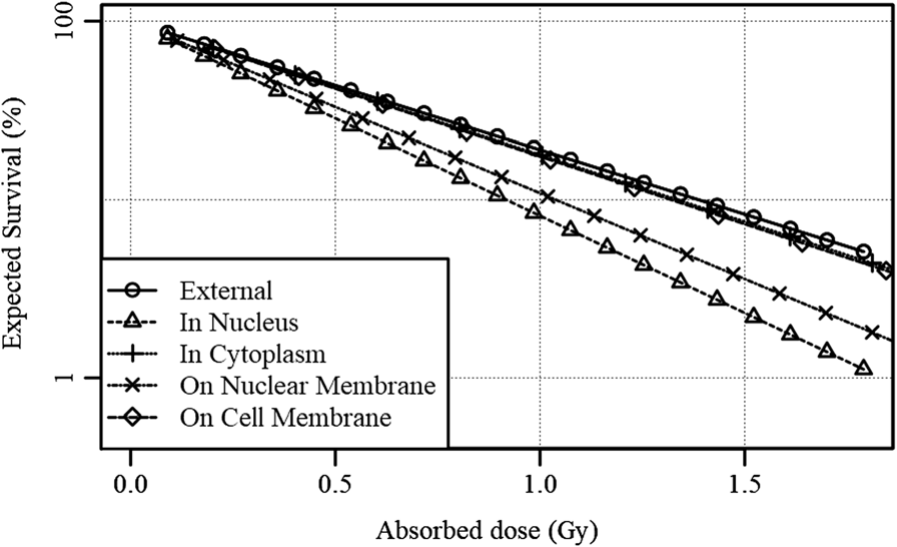
The expected survival as function of absorbed dose for virtual spheroids irradiated internally with Am241 isotopes distributed according to various scenarios, versus an externally irradiated monolayer

2D representations of the distribution of Am241 isotope decay events that yielded an alpha particle that hits the center are shown in Fig. 10. Note that while it was assumed that there was no interstitial fluid in the spheroid, in order to simplify the calculation, the cell membrane for the ‘Isotopes on the cell membrane’-scenario was taken as a sphere with a radius of *r = R*_*eff*_, as given in the Methods section. One can see that the probability of hitting the center nucleus decreases with increasing distance, as is expected from the r-square law. Using the SHNDD calculated for the given scenarios together with *α*_*z*_ = 1.92, the expected survival displayed in Fig. 11 could be calculated. Note that RBE effects due to differences in LET between the different scenarios are ignored in these calculations. It was found that for low energy alpha particles, in some scenarios, a notable difference in absorbed dose in the nucleus and the cytoplasm was present. The nucleus – cytoplasm dose ratios for Am241 isotopes distributed according to the described scenarios are shown in.

Table 1, together with the relative effectiveness (without RBE contribution), given by the ratio of survival slopes obtained from Fig. 11. The average LET in the cell nuclei and the LET-based RBE relative to the external irradiation [34] are shown as well for comparison. Note that Tracy et al. [28] reported a saturation effects for LETs above 130 keV, which lead to an average RBE in the spheroid calculations below that of the external radiation.

**Table 1:** Nucleus – cytoplasm dose ratio together with the relative effectiveness (found in Fig. 11), the average LET and the relative LET-based RBE compared to the external irradiation for Am241 isotopes in virtual spheroid according to various spatial distributions of isotopes

Isotope distribution:	In Nucleus	In Cytoplasm	On Nuclear membrane	On Cell Membrane
Nucleus – Cytoplasm dose ratio	140%	94%	125%	95%
Relative effectiveness ^a^	151%	104%	133%	105%
Average LET (keV/μm)	102 ± 36	128 ± 38	104 ± 49	129 ± 37
LET-based RBE ^a^	0.91	0.93	0.85	0.93

^a^ relative to the external irradiation

The differences in expected survival from the NDD for isotopes in the cytoplasm and on the cell membrane versus the externally irradiated monolayer can reasonably be expected to be within the margin of error of these simulations. However, isotopes in the cell nucleus and on the nuclear membrane show a substantial difference in expected survival with relative effectivenesses of respectively 151% and 133%. Considering the contributing (radio) biological factors found in spheroids like the Oxygen Enhancement Ratio [35, 36] (OER), cell cycle changes and accompanying radiosensitivity [37, 38], and the difficulty of accurate survival studies in spheroids, NDD-based microdosimetry has only a marginal effect on the survival for most ARCs in spheroids and micro-tumors based on the presented data.. From these results it can be concluded that accurate determination of OER and cell cycle and LET based RBE effects should precede NDD-based microdosimetry in the process of translating monolayer survival experiments to the expected survival in spheroids and micro-tumors.

**Fig. 12:**
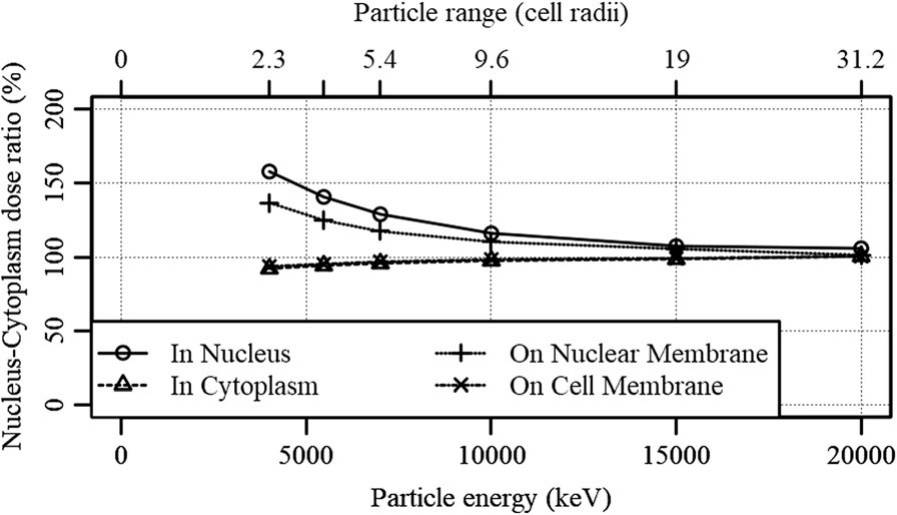
Nucleus – cytoplasm dose ratio as function of particle energy. The respective range of the particle in water is given as the range in number of U87 cell radii

In order to explore the limits of the effects of NDD-based microdosimetry, the nucleus – cytoplasm dose ratio was calculated for imaginary alpha emitting isotopes with alpha particle energies between 4000 and 20,000 keV. Figure 12 shows a distinct relation between the nucleus – cytoplasm dose ratio and the initial particle energy. Since a higher energy translates to a longer range in water and a lower initial LET, the dose deposited by single particle becomes less localized with increasing energy. A similar decrease in relative effectiveness was seen for the expected survival as function of particle energy. The nucleus – cytoplasm dose ratio reached values within the experimental error for particle ranges between 10 and 20 cell radii in all 4 scenarios. This is however above the maximum alpha particle energy in use (8784 keV, Po212 [39]) when using the U87 cell line used in these experiments. It is therefore expected that ARCs attached to, or penetrating, the nuclear membrane will benefit from NDD-based microdosimetry, especially considering the contribution of recoiling daughter radionuclides, an effect that was not included in these calculations [3].

## Conclusion

It was shown that the individual alpha tracks measured using FNTDs can be applied for microdosimetric purposes. By using 3D scans of the investigated cells, microdosimetric spectra could be obtained with great detail and with relative easy. While only small discrepancies were observed between the analytical and experimental approach for NDD estimation as indicated by the small RMSE, validation of the obtained NDD was not yet possible due to a lack of comparable tools for NDD estimation. Since no assumptions regarding the radiation field or target geometry were required, the method is expected to be more reliable and easier compared to analytical or Monte Carlo approaches. The FNTD approach has potential to be of great tool for DNA damage and repair studies with alpha radiation, since FNTDs offer, in addition to the fluorescent tracks, a biocompatible surfaces on which live cells can be grown, irradiated and imaged [40]. The multi-cell approach proved to be a good estimator for the NDD and the survival of the whole cell culture, and allowed for calculation of *α*_*z*_ as function of the measured survival parameter *α*_*D*_, making this approach the first of its kind to do so. Simulations of Am241 isotopes in the virtual U87 spheroids showed that the NDD- has a marginal effect on the expected survival for ARCs in the cytoplasm and on the cell membrane compared to other contributing factors found in the dosimetry and radiobiology of spheroids and micro-tumors. A substantial difference in both expected survival and nucleus-cytoplasm dose ratio was observed for isotopes in the cell nucleus and on the nuclear membrane. These effects were found to vanish for alpha particles with ranges in water from 10 to 20 cell radii. However, since these ranges corresponds to alpha particle energies between 10.000 and 16.000 keV, which are above the maximum alpha particle energy of alpha particle emitting radionuclides (8784 keV, Po212 [39]), it is expected that ARCs that attach to, or penetrate, the nucleus membrane will benefit from specific energy distribution-based microdosimetry.

## Abbreviations

Am241: Americium-241
ARC: Alpha Radionuclide Carrier
FNTD: Fluorescent Nuclear Track Detector
HCP: Hexagonal Close Packing
LET: Linear Energy Transfer
NDD: Nucleus Dose Distribution
OER: Oxygen Enhancement Ratio
RBE: Relative Biological Effectiveness
RMSE: Root-mean-square error
SHNDD: Single Hit Nucleus Dose Distribution
